# The Impact of Different Nursing Skill Mix Models on Patient Outcomes in a Respiratory Care Center

**DOI:** 10.1111/j.1741-6787.2012.00246.x

**Published:** 2012-12

**Authors:** Pei-Hsuan Yang, Chich-Hsiu Hung, Yao-Mei Chen, Chuan-Yu Hu, Sheng-Lan Shieh

**Affiliations:** School of Nursing, Kaohsiung Medical UniversityKaohsiung, Taiwan and Head Nurse, Department of Nursing, Kaohsiung Medical University HospitalKaohsiung, Taiwan

**Keywords:** respiratory care, nursing skill mix models, patient outcomes

## Abstract

**Background:**

Many hospitals have reformed hospital policies and changed nursing models to cope with shortages in nursing staff and control medical costs. However, the nursing skill mix model that most successfully achieves both cost effectiveness and quality care has yet to be determined.

**Aim:**

The aim of this study was to explore the impact of different nurse staffing models on patient outcomes in a respiratory care center (RCC).

**Methods:**

Retrospective data from 2006 to 2008 were obtained from records monitoring nursing care quality, as well as patient records and nursing personnel costs in an RCC as a medical center, in southern Taiwan. A total of 487 patients were categorized into two groups according to the RCC's mix of nursing staff. The “RN/Aide” group comprised 247 patients who received RN and aide care, with a 0.7–0.8 proportion of RNs, from July 2006 to June 2007. The other 240 patients (“All-RN”) received 100% RN care from January 2008 to December 2008.

**Results:**

The results of this study indicated no significant differences in occurrence of pressure ulcer or respiratory tract infections, days of hospitalization, mortality, or nursing costs. However, significant differences were observed in ventilator weaning and occurrence of urinary tract and bloodstream infections.

**Conclusions:**

A higher proportion of RNs was associated not only with a lower rate of urinary tract infection but also with more patients being weaned successfully from ventilators. The findings of this study have implications for how managers and administrators manage nurse staffing in respiratory care.

## BACKGROUND

Statistics from the Bureau of National Health Insurance (BNHI) in Taiwan revealed that the 11,578 patients who were on ventilators for more than 21 days and issued catastrophic illness cards in 1999 cost 3.5 billion New Taiwan Dollars (NTD) in medical expenses (BNHI 2000). The BNHI therefore launched the “Integrated Delivery System (IDS) Demonstration for Ventilator-Dependent Patients” in 2000, in which different nursing care and payment methods were applied to four levels of respiratory care: the intensive care unit (ICU), the respiratory care center (RCC), the respiratory care ward (RCW), and home care. In addition to enhancing ICU bed utilization, the IDS was designed and expected to reduce costs, apply medical resources effectively, and maintain good quality of care.

In recent years, hospital quality management has focused on outcome-oriented indicators (Taiwan Joint Commission on Hospital Accreditation 2010). Patient outcomes include nosocomial infections, ventilator weaning, pressure ulcers, falls, mortality, and days of hospitalization, among which health care-associated infections and mortality are considered important indicators of health care (Cho et al. 2003; Currie et al. 2005; McCloskey & Diers 2005; BNHI 2009; Taiwan Joint Commission on Hospital Accreditation 2010). Orrett (2002) analyzed data obtained from 629 ICU inpatients and discovered that the respiratory tract (29.5%), surgical wounds (25.2%), and urinary tract (20.1%) were the most common sites of nosocomial infection. Nosocomial pneumonia among such patients was common and associated with the use of ventilators (87.4%), resulting in higher mortality rates and more days of hospitalization than nonventilator-associated pneumonia (Suka et al. 2007). Patients without infections had a higher ventilator-weaning rate and lower mortality rate than patients with infections. Aboussouan et al. (2008) assessed the factors affecting mortality among 117 patients in a respiratory unit and found that patients older than age 65 who developed pressure ulcers and failed to be weaned from the ventilator had a higher rate of mortality.

Nurse staffing is believed to affect patient outcomes. Su et al. (2010) concluded that nurse staffing was significantly related to patient safety, infection rate, and mortality. In a study exploring 51 ICUs within 31 hospitals, Stone et al. (2007) noted a significant correlation between nurse staffing and the development of bloodstream infection, ventilator-associated pneumonia, urinary tract infection, and pressure ulcers; sufficient nurse staffing could effectively reduce the occurrence of infection (Stone et al. 2007). To cope with nursing staff shortages, medical institutions have deployed skill mix models to recruit nursing aides to care for patients and assist with various unit duties under the supervision and guidance of nurses (Mei et al. 2009).

A study concerning the relationship of skill mix models to patient outcomes by Needleman et al. (2002) showed that when patients received more hours of care from registered nurses (RN) compared to hours of care from nursing aides, they had fewer days of hospitalization as well as lower rates of urinary tract infection, gastric hemorrhage, pneumonia, shock, and cardiac arrest, indicating that RNs provided a better quality of nursing care. Yang (2003) conducted a retrospective study of 21 patients in the internal medicine and surgical wards of a medical center and analyzed data obtained from records monitoring nursing care quality, patient records, a patient classification system, and nurse staffing. Results indicated that nursing workload was the most important predictor of nosocomial infections; patients developing nosocomial infections increased the cost per patient to approximately 115,710 NTD and added 18.2 days of hospitalization (Chen et al. 2005). In sum, the idea of decreasing nursing staff to save personnel costs could lead to prolonged hospital stays, higher medical costs, and increased risk of life-threatening conditions.

Based on BNHI regulations, the nurse-to-patient ratio should be 1:1; however, some hospitals employ nursing aides to perform nonprofessional nursing duties as a means of reducing nurse-staffing costs. In 2003, the Taiwanese government allocated US $6 million to train and employ nursing aides (Tzeng 2004) and then, in 2005, planned skill mix models in which specific job duties were assigned to RNs and to nursing aides (Lu 2009). By successfully completing a 90-hour training course, return demonstration, and clinical practicum offered by the Department of Health, Executive Yuan, Taiwan, nursing aides could qualify to assist patients in bathing, turning, and feeding, taking on some of the tasks usually assigned to RNs.

The government has already spent large amounts of money and resources in training nursing aides, yet studies about the effects of skill mix models on patient outcomes have been limited to general wards (You et al. 2003; Feng et al. 2008), and few related studies have been conducted in progressive care units or ICUs.

### Purpose

Seeking further understanding of the impact of nurse staffing on patient outcomes, our research examined the following: (1) differences in patient outcomes (pressure ulcers, respiratory and urinary tract infections, bloodstream infections, days of hospitalization, and ventilator weaning) between patients who received “All-RN care” and patients who received “RN/Aide care”; and (2) differences in staffing costs between the two aforementioned groups.

## METHODS

### Participants and Setting

The mixed RN/Aide assignment in a medical center in southern Taiwan was initiated in January 2003. However, the manpower for nursing aides was unstable until July 1, 2006. Thus, a total of 487 patients referred to an RCC at the medical center from July 1, 2006, to December 31, 2008, were categorized into two groups according to the mix of nursing staff: 247 patients were cared for by both RNs and nursing aides (“RN/Aide care,” substituting one RN with two nursing aides, with the proportion of RNs = 0.6–0.7) from July 1, 2006, to June 30, 2007; 240 were cared for by RNs only (“All-RN care,” proportion of RNs = 1) from January 1 to December 31, 2008. The proportion of RNs was defined as the percentage of RNs to total nurse staffing (RNs and nursing aides). In consideration of staffing costs, the human alternative of one RN was two nursing aides.

### Data Collection Procedures

The study protocol was approved by the Institutional Review Board of the Kaohsiung Medical University Hospital. Retrospective patient data and nursing staff records were collected from July 1, 2006, to June 30, 2007, and from January 1 to December 31, 2008, during which time patients were referred to the RCC and received respiratory therapy.

Demographic data obtained from patient records included age, sex, the Acute Physiology and Chronic Health Evaluation II (APACHE II), tracheostomy, underlying diseases, days of ventilator use, days of hospitalization in the RCC, and records of ventilator use. APACHE II is a severity-of-disease classification system. Within 24 hours of admission to an RCC, a patient's integer score was computed based on several measurements (temperature, heart rate, mean arterial pressure, respiratory rate, PaO_2_, serum sodium, white blood cell count, platelet count, Glasgow coma score, and marks adjusting for patient age and for chronic health problems). Higher APACHE II scores imply more severe disease and higher risk of death (Knaus et al. 1985).

Patient-outcome data obtained from infection control, quality management, and human resource departments included pressure ulcers, respiratory and urinary tract infections, bloodstream infections, ventilator weaning, mortality, and nursing cost per patient in the RCC. During the period of this study, the conditions of pressure ulcers, respiratory and urinary tract infections, and bloodstream infections were recorded after each patient's admission to an RCC. Successful ventilator weaning is defined by BNHI as liberation from mechanical ventilation for more than 72 consecutive hours.

Nursing costs were defined as the monetary amount spent by the RCC to provide service to the patients during the study period. Nursing cost data were provided by the hospital accounting office. For this study, we computed the 12-month nursing cost for both RN/Aide and All-RN care groups. The 12-month nursing cost for each group was divided by 12 months to determine an average nursing cost per month for the two groups.

### Data Analysis

Data were encoded and repeatedly verified for accuracy and then analyzed using JMP 8.0 statistical software and adequate statistical methods. Mean and standard deviation were used to describe continuous variables of patients’ demographic data; frequency distribution and percentage were used to present categorical variables. The χ^2^ test and *t*-test were used to compare the differences between the two groups in the occurrence of pressure ulcers, respiratory and urinary tract infections, bloodstream infections, days of hospitalization in the RCC, ventilator weaning, mortality, and nursing costs.

## FINDINGS

### Participants’ Demographic Characteristics

The mean ages of the patients in the two groups were 67.80 ± 15.74 years and 70.09 ± 14.31 years, respectively; males, 145 (58.70%) and 130 (54.17%), respectively, were in the majority; the APACHE II scores were 16.13 ± 5.62 and 17.03 ± 5.73, respectively; 135 (54.66%) and 128 (53.33%) had previously undergone a tracheostomy when referred to the RCC; 74 (29.96%) and 91 (37.92%) had respiratory disease (chronic lung disease following acute lung injury), which was the most commonly occurring underlying disease, followed by neuromuscular disorder (intracerebral hemorrhage), 73 (29.55%) and 68 (28.33%). The mean lengths of ventilator use in the RCC were 19.33 ± 13.80 and 17.60 ± 12.57 days; the mean lengths of ventilator use during total hospitalization were 40.04 ± 15.42 and 39.99 ± 15.92 days (Table 1). Statistics indicated that no significant differences existed in the demographic characteristics between the All-RN and RN/Aide groups.

**TABLE 1:** Patients' demographic characteristics between two different nursing staff models (*n*= 487)

	NURSE AND AIDE (*n*= 247)	NURSE (*n*= 240)		
				
VARIABLES	*n*(%) or MEAN ± SD	*n*(%) or MEAN ± SD	χ^2^/*t*	*P*
Age	67.80 ± 15.74	70.09 ± 14.31	1.68	0.09
Gender				
Male	145 (58.70)	130 (54.17)	1.02	0.31
Female	102 (41.30)	110 (45.83)	–	–
APACHE II	16.13 ± 5.62	17.03 ± 5.73	1.74	0.08
Tracheostomy				
Yes	135 (54.66)	128 (53.33)	0.09	0.77
No	112 (45.34)	112 (46.67)	–	–
Underlying disease				
Lung disease	74 (29.96)	91 (37.92)	4.84	0.57
Postsurgery	10 (4.05)	6 (2.50)	–	–
Neuromuscular disease	73 (29.55)	68 (28.33)	–	–
Cardiac disease	27 (10.93)	22 (9.17)	–	–
Sepsis and other	36 (14.60)	27 (11.25)	–	–
Cancer	27 (10.93)	26 (10.83)	–	–
Days for ventilator in RCC	19.33 ± 13.80	17.60 ± 12.57	1.44	0.15
Days for ventilator during hospitalization	40.04 ± 15.42	39.99 ± 15.92	0.03	0.97

*Note*. APACHE II = the Acute Physiology and Chronic Health Evaluation II; *P > 0.05 = no significance.

### Comparisons of Patient Outcomes and Nursing Costs

In terms of patient outcomes, no significant difference existed in the occurrence of pressure ulcers and respiratory infections, the mean lengths of RCC stay, mortality, or nursing costs. Yet significant differences existed in urinary tract and bloodstream infections (*t*= 24.88, *P* < 0.01; *t*= 8.47, *P*= 0.01), indicating that patients in the All-RN group had fewer urinary tract infections than those in the RN/Aide group (6.67% compared to 22.26%), although patients in the All-RN group had more bloodstream infections than those in the RN/Aide group (12.92% compared to 6.88%). Patients in the All-RN group also had a significantly higher rate of ventilator weaning (χ^2^= 10.85, *P* < 0.01; Table 2).

**TABLE 2:** Patient outcomes and nursing cost between different nursing staffs (*n*= 487)

	NURSE AND AIDE *n*= 247	NURSE *n*= 240		
				
VARIABLES	*n*(%) or MEAN ± SD	*n*(%) or MEAN ± SD	χ^2^*/t*	*P*
Pressure ulcer	3 (1.21)	7 (2.92)	1.75	0.19
Urinary tract infections	55 (22.26)	16 (6.67)	24.88	<0.01**
Bloodstream infections	17 (6.88)	31 (12.92)	8.47	0.01**
Respiratory tract infections	8 (3.24)	8 (3.33)	0.003	0.95
Days of hospitalization in RCC	22.29 ± 12.54	21.69 ± 12.03	0.54	0.59
Ventilator weaned			10.85	<0.01**
Yes	92 (37.25)	125 (52.08)		
No	155 (62.75)	115 (47.92)		
Mortality	50 (20.24)	45 (18.75)	0.17	0.68
Nursing cost per month (NT)	1574,489.00 ± 112,022.00	1571,054.00 ± 142,722.00	0.07	0.95

NT = New Taiwan dollar;

** *P* < 0.01.

## DISCUSSION

Evidence shows that nurse staffing affects patient safety and the quality of nursing care, and that nursing professionals contribute to human health. Previous studies have pointed out that patient outcomes such as mortality, pressure ulcers, and nosocomial infections increase when the number of patients for each nurse increases; in other words, each additional nurse reduces patient mortality by 1.2% (Aiken et al. 2002; Kane et al. 2007). However, our study presented no significant mortality difference between different nurse staffing models; the reason for this could be associated with the participants we recruited for this study. Most patients who are referred to an RCC are elderly (65 years old or older) and have multiple chronic diseases, chronic kidney disease, coronary artery disease, and other complications (Su et al. 2006; Yang et al. 2008). Therefore, the study findings show no significant difference in mortality between different nurse staffing models.

Our findings indicate that a higher proportion of RNs could reduce the occurrence of urinary tract infection in patients, which is in line with Needleman et al. (2002) in their study of the relationship between nurse staffing levels and quality of care. However, our study revealed that more bloodstream infections occurred in the group with the higher proportion of RNs. This is a different conclusion than that reached by Kane et al. (2007), who explored the association between nurse staffing levels and bloodstream infections in acute care hospitals, and found that each additional nurse reduces bloodstream infections in patients by 36%. The reason for this may be attributed to the participants’ characteristics.

Most patients referred to an RCC have multiple diseases (Yang et al. 2008), and the retention time of most patients’ central venous catheters (CVCs) was longer than 14 days. In a study surveying the prevention of catheter-related infections, Mermel (2000) found that 90% of bloodstream infections are associated with the retention of CVCs. Byrnes and Coopersmith (2007) found that hand hygiene is one of the most important preventive strategies for bloodstream infection. In our study, patients who received RN/Aide care were given bed baths by the nursing aides and were then cared for by RNs, who inserted CVCs and addressed wound care at insertion sites. However, patients who received All-RN care were given bed baths and received care from RNs, including CVC insertions and wound care at insertion sites. According to the author's regular observations of care, some of these RNs might fail to follow the standard procedure of performing catheter care only after changing gloves instead of washing their hands. This failure could explain why the patients who received All-RN care developed more bloodstream infections than those patients who received RN/Aide care. Thus, in-service education for basic aseptic technique and standard practice procedure must be emphasized and regularly monitored.

In the Taiwanese medical system, nursing staff are faced with a heavy burden of duties to meet the accreditation criteria of high quality of nursing care, along with a nursing shortage and high turnover rate. It is essential that nursing staff have enough time to perform professional duties by recruiting nursing aides to reduce nurses’ workload. However, how to apply nursing aides without sacrificing quality of care remains a major issue.

In-service education should be conducted to teach nursing staff the role and function of nursing aides, which may help them understand how to effectively instruct and supervise aides in performing standard nursing skills. Moreover, skill mix nursing models should be included in school nursing curricula to help nursing students gain a better understanding of the role and function of nursing aides and learn to apply nursing aides effectively in patient care, leaving them adequately prepared for future clinical practice.

Our study also discovered that different nurse staffing models affect patients’ weaning from ventilators. More patients who received All-RN care were successfully weaned from their ventilators compared to patients who received RN/Aide care. Moreover, fewer patients who received All-RN care failed to be weaned from the ventilator compared to the patients who received RN/Aide care. As noted in previous qualitative studies, the concern of nurses and close family members for patients is an important factor in the process of being weaned from the ventilator (Chen et al. 2009). Our findings support the notion that conscious patients need more psychological support, and that an increase in professional nursing staff to guide the weaning process could effectively reduce patients’ fear and stress. RNs are able to observe a patient's respiratory intolerance during the weaning process and impart their medical knowledge, experience, and patience to the patient, bolstering the patient's sense of security and confidence in detaching from the ventilator.

This study was conducted with a retrospective chart review from July 1, 2006, to June 30, 2007, and from January 1 to December 31, 2008, during which time patients were referred to an RCC and received respiratory therapy. A possible limitation of the study is that the patients in the two groups were treated in different historical contexts within 12-month time periods. The structural change and temporally contingent relations that occurred in the different periods could not be quantitatively analyzed.

In this study, nursing costs did not significantly differ between the two groups. This could be anticipated because of the payment of two nursing aides and one RN balancing each other out. However, findings suggest an association between nurse staffing and patient outcomes, and the findings mirror those found in the study of Needleman et al. (2002) on the relationship between nurse staffing levels and quality of patient care, and those of Su et al. (2010) on the relationship between nurse staffing and patient outcomes. According to IDS regulations for ventilator-dependent patients, a patient who has been weaned successfully from the ventilator and closely observed for 5 days must be referred to a general ward, home care, or a nursing home for long-term care. Therefore, the improved ventilator weaning of patients receiving All-RN care observed in this study suggests that care exclusively by RNs may reduce the length of hospitalization in the RCC.

The aging of Taiwan's population has led to an increase in chronic diseases as well as in the number of ventilator-dependent patients who need respiratory care. Because of medical cost constraints, a number of medical institutions have reduced the number of RNs they employ and instead hired nursing aides in an effort to cut nursing personnel costs. Our study reveals that substituting RNs with nursing aides increases urinary tract infections and reduces the success rate of weaning patients from ventilators, which could result in increased medical costs. Currently, in Taiwan, nursing aides are trained by an external agency. Medical institutions could also explore training nursing aides based on their own institutional care standards and compare patient outcomes between two different skill mix nursing models with different training processes.

## CONCLUSIONS

Nurse staffing is a complex policy issue, and various inventive nursing models have been introduced in attempts to reduce the turnover rate of nurses in Taiwan. The primary nursing, magnet hospital, and nursing skill mix models have been promoted since 2005 (Tsay & Wang 2007), yet the nurse turnover rate remains high and has reached 46.5% (Lee et al. 2009). According to the International Council of Nurses (2006), factors affecting the supply and demand for nurses include number of nurses, nursing skill mix models, case mix complexity, and cost effectiveness. In addition to improved nurse education, future studies are necessary to explore the relationships between different nursing models and patient outcomes, and to identify an appropriate nursing model that can stabilize a nursing workforce without sacrificing quality of care or overly increasing nursing cost.

## References

[b1] Aboussouan LS, Lattin CD, Kline JL (2008). Determinants of long-term mortality after prolonged mechanical ventilation. Lung.

[b2] Aiken LH, Clarke SP, Sloane DM, Sochalski J, Silber JH (2002). Hospital nurse staffing and patient mortality, nurse burnout, and job dissatisfaction. Journal of the American Medical Association.

[b4] Bureau of National Health Insurance (2000). http://www.nhi.gov.tw/webdata/webdata.aspx?menu=17&menu_id=661&WD_ID=689&webdata_id=3351.

[b3] Bureau of National Health Insurance (2009). National Health Insurance ventilator-dependent patients with integrated care prospective payment pilot project *(approved version)*.

[b5] Byrnes MC, Coopersmith CM (2007). Prevention of catheter-related blood stream infection. Current Opinion in Critical Care.

[b6] Chen CJ, Lin CJ, Tzeng YL, Hsu LN (2009). Successful mechanical ventilation weaning experiences at respiratory care centers. The Journal of Nursing Research.

[b7] Chen YY, Chou YC, Chou P (2005). Impact of nosocomial infection on cost of illness and length of stay in intensive care units. Infection Control and Hospital Epidemiology.

[b8] Cho SH, Ketefian S, Barkauskas VH, Smith DG (2003). The effects of nurse staffing on adverse events, morbidity, mortality, and medical costs. Nursing Research.

[b9] Currie V, Harvey G, West E, McKenna H, Keeney S (2005). Relationship between quality of care, staffing levels, skill mix and nurse autonomy: Literature review. Journal of Advanced Nursing.

[b10] Feng RC, Sang YY, Chang WC, Chen MM, Chou SO, Yin JC (2008). Establishment and evaluation of the skill-mix model in a medical center. Veterans General Hospital Nursing.

[b11] International Council of Nurses (2006). Safe staffing saves lives: Information and action tool kit.

[b12] Kane RL, Shamliyan TA, Mueller C, Duval S, Wilt TJ (2007). The association of registered nurse staffing levels and patient outcomes: Systematic review and meta-analysis. Medical Care.

[b13] Knaus WA, Draper EA, Wagner DP, Zimmerman JE (1985). APACHE II: A severity of disease classification system. Critical Care Medicine.

[b14] Lee TY, Tzeng WC, Lin CH, Yeh ML (2009). Effects of a preceptorship program on turnover rate, cost, quality and professional development. Journal of Clinical Nursing.

[b15] Lu MS, Lu MS (2009). Discussion on professional nursing issues. Delivery integrated care model: Technical feasibility of mixed model.

[b18] McCloskey BA, Diers DK (2005). Effects of New Zealand's health reengineering on nursing and patient outcomes. Medicine Care.

[b17] Mei TT, Lee JL, Liang YW, Liu LF, Huang LC (2009). Exploring the current state and progress of the skill-mix model in Taiwan. The Journal of Nursing.

[b16] Mermel LA (2000). Prevention of intravascular catheter- related infections. Annals of International Medicine.

[b19] Needleman J, Buerhaus P, Mattke S, Stewart M, Zelevinsky K (2002). Nurse-staffing levels and the quality of care in hospitals. New England Journal of Medicine.

[b20] Orrett FA (2002). Nosocomial infections in an intensive care unit in a private hospital. West Indian Medical Journal.

[b21] Stone PW, Mooney-Kane C, Larson EL, Horan T, Glance LG, Zwanziger J, Dick AW (2007). Nurse working conditions and patient safety outcomes. Medical Care.

[b22] Su J, Lin CY, Chen PJ, Lin FJ, Chen SK, Kuo HT (2006). Experience with a step-down respiratory care center at a tertiary referral medical center in Taiwan. Journal of Critical Care.

[b23] Su SF, Liu PE, Su HY (2010). The relationships among the nurse staffing, negative patient outcomes, and mortality. Cheng Vhing Medical Journal.

[b24] Suka M, Yoshida K, Uno H, Takezawa J (2007). Incidence and outcomes of ventilator-associated pneumonia in Japanese intensive care units: The Japanese nosocomial infection surveillance system. Infection Control and Hospital Epidemiology.

[b25] Taiwan Joint Commission on Hospital Accreditation (2010). Taiwan Clinical Performance Indicator.

[b26] Tsay SF, Wang HH (2007). Reflections on nursing education in Taiwan and its prospects from the perspective of nursing manpower policy. The Journal of Nursing.

[b27] Tzeng HM (2004). Roles of nurse aides and family members in acute patient care in Taiwan. Journal of Nursing Care Quality.

[b28] Yang KP (2003). Relationships between nurse staffing and patient outcomes. The Journal of Nursing Research.

[b29] Yang PH, Hung JY, Yang CJ, Tsai JR, Wang TH, Lee JC, Huang MS (2008). Successful weaning predictors in a respiratory care center in Taiwan. The Kaohsiung Journal of Medical Sciences.

[b30] You LH, Chen CF, Cheng TM, Wu WS (2003). Taiwan hospital culture reform—cultural change in Taiwan's hospitals full responsibility for care of Taipei City Hospital, the pilot project.

